# Molecular Basis of Rare Aminoglycoside Susceptibility and Pathogenesis of *Burkholderia pseudomallei* Clinical Isolates from Thailand

**DOI:** 10.1371/journal.pntd.0000519

**Published:** 2009-09-22

**Authors:** Lily A. Trunck, Katie L. Propst, Vanaporn Wuthiekanun, Apichai Tuanyok, Stephen M. Beckstrom-Sternberg, James S. Beckstrom-Sternberg, Sharon J. Peacock, Paul Keim, Steven W. Dow, Herbert P. Schweizer

**Affiliations:** 1 Department of Microbiology, Immunology and Pathology, Rocky Mountain Regional Center of Excellence for Biodefense and Emerging Infectious Diseases Research, Colorado State University, Fort Collins, Colorado, United States of America; 2 Faculty of Tropical Medicine, Mahidol University, Bangkok, Thailand; 3 The Microbial Genetics and Genomics Center, Northern Arizona University, Flagstaff, Arizona, United States of America; 4 Pathogen Genomics Division, Translational Genomics Research Institute, Flagstaff, Arizona, United States of America; 5 Department of Medicine, University of Cambridge, Addenbrooke's Hospital, Cambridge, United Kingdom; Institut Pasteur, France

## Abstract

**Background:**

*Burkholderia pseudomallei* is intrinsically resistant to aminoglycosides and macrolides, mostly due to AmrAB-OprA efflux pump expression. We investigated the molecular mechanisms of aminoglycoside susceptibility exhibited by Thai strains 708a, 2188a, and 3799a.

**Methodology/Principal Findings:**

qRT-PCR revealed absence of *amrB* transcripts in 708a and greatly reduced levels in 2188a and 3799a. Serial passage on increasing gentamicin concentrations yielded 2188a and 3799a mutants that became simultaneously resistant to other aminoglycosides and macrolides, whereas such mutants could not be obtained with 708a. Transcript analysis showed that the resistance of the 2188a and 3799a mutants was due to upregulation of *amrAB-oprA* expression by unknown mechanism(s). Use of a PCR walking strategy revealed that the *amrAB-oprA* operon was missing in 708a and that this loss was associated with deletion of more than 70 kb of genetic material. Rescue of the *amrAB-oprB* region from a 708a fosmid library and sequencing showed the presence of a large chromosome 1 deletion (131 kb and 141 kb compared to strains K96243 and 1710b, respectively). This deletion not only removed the *amrAB-oprA* operon, but also the entire gene clusters for malleobactin and cobalamin synthesis. Other genes deleted included the anaerobic arginine deiminase pathway, putative type 1 fimbriae and secreted chitinase. Whole genome sequencing and PCR analysis confirmed absence of these genes from 708a. Despite missing several putative virulence genes, 708a was fully virulent in a murine melioidosis model.

**Conclusions/Significance:**

Strain 708a may be a natural candidate for genetic manipulation experiments that use Select Agent compliant antibiotics for selection and validates the use of laboratory-constructed Δ(*amrAB-oprA*) mutants in such experiments.

## Author Summary

*Burkholderia pseudomallei* is the etiologic agent of melioidosis, an emerging tropical disease. Because of low infectious dose, broad-host-range infectivity, intrinsic antibiotic resistance and historic precedent as a bioweapon, *B. pseudomallei* was listed in the United States as a Select Agent and Priority Pathogen of biodefense concern by the US Centers for Disease Control and Prevention and the National Institute of Allergy and Infectious Diseases. The mechanisms governing antibiotic resistance and/or susceptibility and virulence in this bacterium are not well understood. Most clinical and environmental *B. pseudomallei* isolates are highly resistant to aminoglycosides, but susceptible variants do exist. The results of our studies with three such variants from Thailand reveal that lack of expression or deletion of an efflux pump is responsible for this susceptibility. The large deletion present in one strain not only removes an efflux pump but also several putative virulence genes, including an entire siderophore gene cluster. Despite this deletion, the strain is fully virulent in an acute mouse melioidosis model. In summary, our findings shed light on mechanisms of antibiotic resistance and pathogenesis. They also validate the previously advocated use of laboratory-constructed, aminoglycoside susceptible efflux pump mutants in genetic manipulation experiments.

## Introduction

Melioidosis is a disease caused by *Burkholderia pseudomallei* [1],[2]. Melioidosis is endemic to tropical and subtropical regions of the world [3] and is considered an emerging disease (e.g. NE Thailand [4]) as well as a disease of biodefense importance [4]. Melioidosis has received worldwide popular attention in the wake of the 2004 SE Asia Tsunami disaster [5],[6],[7],[8]. Treatment of melioidosis is complicated by the intrinsic resistance of *B. pseudomallei* to many antibiotics, including aminoglycosides, macrolides, several penicillins, and first and second generation cephalosporins [1],[2],[9]. Factors complicating drug therapy are the ability of *B. pseudomallei* to form biofilms [10] and to enter into prolonged, presumably intracellular, latency periods of up to six decades in a human host [11].

Genome sequence analysis has provided an indication of possible mechanisms of resistance to antimicrobial compounds, but less than a handful of resistance genes have been experimentally confirmed to date [12]. The K96243 and other *B. pseudomallei* genomes encode an arsenal of efflux pumps, including 10 pumps belonging to the resistance nodulation cell division (RND) family, which play major roles in clinically significant antibiotic resistance in Gram-negative bacteria. Two of these, AmrAB-OprA [13] and BpeAB-OprB [14] were reported to play major roles in high-level resistance to aminoglycosides and macrolides, but our unpublished results with strain 1026b indicate that BpeAB-OprB does not efflux aminoglycosides. Using a surrogate *Pseudomonas aeruginosa* strain we recently showed that BpeEF-OprC extrudes chloramphenicol and trimethoprim [15]. While the majority of clinical *B. pseudomallei* isolates exhibit high levels of aminoglycoside and macrolide resistance, rare (∼1∶1000) isolates are susceptible to these antibiotics. It has been noted that the resistance profile of these isolates matches that of *amrAB-oprA* mutants suggesting possible involvement of AmrAB-OprA in intrinsic aminoglycoside and macrolide resistance or lack thereof [16], but this has not yet been experimentally demonstrated. In this report we provide evidence that the susceptibility of three isolates from NE Thailand is indeed due to lack of, or greatly reduced, AmrAB-OprA expression, either due to deletion or as to as yet unknown mechanisms. Furthermore, deletion of an ∼131 kb region of chromosome 1 in one strain not only removed *amrAB-OprA*, but also genes encoding several putative virulence factors and other functions implicated in bacterial pathogenesis and physiology.

## Materials and Methods

### Bacterial strains, media and growth conditions

*B. pseudomallei* strains used in this study are listed in **Table 1**. *Escherichia coli* strains used for cloning experiments were DH5α [17] or DH5α(λ*pir*) (laboratory strain). All bacteria were routinely grown with aeration at 37°C. Low salt (5 g/L NaCl) Lennox LB broth (LSLB) and agar (MO BIO Laboratories, Carlsbad, CA) were used as rich media. M9 medium [18] with 10 mM glucose was used as the minimal medium. Unless otherwise noted, antibiotics were added at the following concentrations: 100 µg/ml ampicillin (Ap), 12.5 µg/ml chloramphenicol (Cm), 15 µg/ml gentamicin (Gm), 35 µg/ml kanamycin (Km) and 25 µg/ml zeocin (Zeo) for *E. coli*; 1,000 µg/ml Km and 2,000 µg/ml Zeo for wild-type *B. pseudomallei* and 50 µg/ml for Gm susceptible *B. pseudomallei* strains. Antibiotics were either purchased from Sigma, St. Louis, MO (ampicillin, chloramphenicol, erythromycin, kanamycin, polymyxin B and streptomycin), EMD Biosciences, San Diego, CA (gentamicin), Invitrogen, Carlsbad, CA (zeocin) or Biomol via VWR International, West Chester, PA (spectinomycin).

**Table 1:** Strains, plasmids and primers used in this study.

Strain or Plasmid	Relevant Properties^a^	Reference or Source
*B. pseudomallei*		
1026b	AG and ML resistant wild-type strain; clinical isolate	[45]
DD503	AG and ML susceptible Δ(*amrR-amrAB-oprA*)1026b derivative	[13]
708a	AG and ML susceptible clinical isolate	[16]
2188a	AG and ML susceptible clinical isolate	[16]
3799a	AG and ML susceptible clinical isolate	[16]
Bp24	Spontaneous AG and ML resistant derivative of 3799a	This study
Bp35	Spontaneous AG and ML resistant derivative of 2188a	This study
Bp50	1026b with Δ(*amrR-amrAB-oprA*)	[20]
Bp66	Low level Gm^r^ derivative of 708a	This study
Bp187	Bp24 with Δ(*amrR-amrRAB-oprA*)	This study
Bp202	Bp187::mini-Tn*7*T-LAC	This study
Bp194	Bp187::mini-Tn*7*T-LAC-*amrA* ^+^ *B* ^+^-*oprA* ^+^	This study
Bp192	Bp35 with Δ(*amrR-amrAB-oprA*)	This study
Bp201	Bp192::mini-Tn*7*T-LAC	This study
Bp200	Bp192::mini-Tn*7*T-LAC-*amrA* ^+^ *B* ^+^-*oprA* ^+^	This study
*Plasmids*		
pEX-S12*pheS*	Gm^r^; gene replacement vector	Lopez and Schweizer, unpublished
pUC18T-mini-Tn7T-LAC	Ap^r^, Gm^r^; mini-Tn*7* cloning and delivery vector	[46]
pPS2142	Ap^r^, Gm^r^; pUC18T-miniTn*7*T-LAC with *amrA^+^B^+^-oprA^+^*; *amrAB-oprA* expression under *P_tac_* b control	[20]
pTNS3	Ap^r^; source of Tn*7* transposase components TnsABCD	[20]
pFKM2	Ap^r^ Km^r^; source *of FRT*-*nptII*-*FRT* cassette	[20]
pFLPe2^b^	Zeo^r^; source of Flpe recombinase	[20]
pPS1927	Apr; pWSK29 [47] with ∼15 kb strain 1026b chromosomal *Eco*RI fragment containing *amrA^+^B^+^-oprA^+^*	This study
pPS2282	Apr; pGEM-T Easy (Novagen) with ∼3.1 kb PCR fragment containing Δ(*amrAB-OprA*)::*FRT*-*nptII*-*FRT* t	This Study
pPS2354	Gm^r^ Km^r^; pEX-S12*pheS* with ∼3.1 kb blunt-ended *Eco*RI fragment of pPS2282 cloned into the *Sma*I site	This Study
*Primers* c		
597	5′-CGAATTGGGGATCTTGAAGTTCCT	This study
1546	5′-TACATGGCGATAGCTAGACTGG	This study
1599	5′-CGCGCGCAATTGTTCCTC	This study
1600	5′-TCGTAAGAAAGCGACACGCA	This study
1601	5′-CGATTCTTCGCGCGTCTTG	This study
1602	5′-CGCGTGCGTGCCCATTCG	This study
1742	5′-AAGACCGCGCTCTATTACGA	This study
1743	5′-TCGTCACCGTATCAGTGCAT	This study
1756	5′-ATCTTGCCGTTGAAGTGTCC	This study
1757	5′-ATCGCTGAACACGAAGAACC	This study
1774	5′-ACTAGTAGTGAGCGCAACGCAATTA	This study
1779	5′-GCCTCTTCGCTATTACGC	This study
1797	5′-GTTCGTCGCCGAGGAGT	This study
1801	5′-GAAGCCGGTGAAATCGACG	This study
1954	5′-CTCAAGTCGGTGTCCATTCC	This study
1955	5′-ACGTTATCCGGCGTGATCT	This study
2031	5′-CCTGGTTCACCTGCTCGATG	This study
2032	5′-CTTCGTCGCTGCAAGAAACG	This study
2033	5′-CGATCGACCTGCCTGAAACC	This study
2034	5′-AGCTCGTCGTGAACACGGC	This study
2035	5′-GACGTAATGGAACGACGCGC	This study
2036	5′-CGTCGGCGCATTGAACGACA	This study
2037	5′-CGATTCGTACATCGCGGCGA	This study
2038	5′-CTCAACTTCACGGGCGAGAT	This study

a Abbreviations: AG, aminoglycosides; Ap, ampicillin; Gm, gentamicin; Km, kanamycin; ML, macrolides; r, resistance; Zeo, zeocin.

b *P_tac_*, *E. coli lac/trp* operon hybrid promoter.

c Only selected primers are shown; other primer sequences are available from the authors upon request. Oligonucleotides were purchased from IDT, Coralville, IA.

### DNA and genetic methods

Published procedures were employed for manipulation of DNA, and transformation of *E. coli* and *B. pseudomallei* [19],[20],[21]. Plasmid DNAs were isolated from *E. coli* and *B. pseudomallei* using the QIAprep Mini-spin kit (Qiagen, Valencia, CA). Colony PCR with *B. pseudomallei* was performed as previously described [20]. *B. pseudomallei* chromosomal DNA was isolated using the Gentra Puregene DNA purification kit (Qiagen). Custom oligonucleotides were synthesized by Integrated DNA Technologies (Coralville, IA). Isolation of chromosomally-integrated mini-Tn*7* elements followed by Flp-mediated selection marker excision was performed using recently published procedures [20]. Quantitative real-time PCR was performed using the methods and primer sets described by Kumar et al. [22]. Other primer sequences are shown in **Table 1**. Total RNA was extracted from cells grown to late log phase (optical density at 600 nm ∼0.7) in LSLB medium without antibiotics using the RNeasy Mini Kit (Qiagen).

### Mutant construction

For isolation of Δ(*amrR-amrAB-oprA*) mutants, three partially overlapping DNA fragments representing flanking DNA segments and the Km^r^ marker were PCR-amplified from 50 ng pPS1927 and pFKM2 [20] DNA templates and then spliced together by an overlap extension PCR. To do this, the following fragments were amplified in a first-round PCR using Platinum *Taq* HiFi DNA polymerase (Invitrogen, Carlsbad, CA) and the following primers: a 892-bp *amrR* upstream fragment using primers 1581 (5′- agggtgtccacatccttgaa) and 1582 (5′- TCAGAGCGCTTTTGAAGCTAATTCGggacacttcaacggcaagat), a 828-bp *oprA* downstream fragment using primers 1583 (5′- AGGAACTTCAAGATCCCCAATTCGgtcgccgaatacgagaagac) and 1584 (5′- gaaatacgccttgacgcact), and a 1382-bp *FRT*-*nptII*-*FRT* fragment using primers 596 (5′-CGAATTAGCTTCAAAAGCGCTCTGA) and 597 (5′-CGAATTGGGGATCTTGAAGTTCCT)(Lowercase letters denote chromosome-specific sequences and uppercase letters *FRT* cassette-specific sequences.) These fragments were combined in a second PCR and, after gel purification, the resulting recombinant ∼3.1-kb DNA fragment was cloned into pGEM-T Easy (Novagen), which yielded pPS2282. The Δ(*amrR-amrAB-oprA*::*FRT*-*nptII*-*FRT*) cassette was excised from pPS2282 with *Eco*RI, blunted ended with T4 DNA polymerase (NEB) and ligated into the *Sma*I site of pEX-S12*pheS* (C. Lopez and H. Schweizer, unpublished) yielding pPS2354. Gene replacement using PheS-mediated counter-selection on M9-glucose supplemented with 0.15% *p*-chlorophenylalanine was performed as previously described [23] except that *E. coli* strains SM10(λ*pir*) or RHO1 (a Km susceptible derivative of SM10[λ*pir*] [24]; D. Rholl and H. Schweizer, unpublished) were used for conjugation experiments. The recipient strain was either Bp24 or Bp35 and merodiploids were selected on LSLB medium supplemented with 1000 µg/ml Km (to select for the Δ[*amrR-amrAB-oprA*::*FRT*-*nptII*-*FRT*] cassette cloned in pEX-S12*pheS*) and 100 µg/ml polymyxin B (to counterselect against RHO1). *p*-chlorophenylalanine resistant colonies were then obtained and screened for the presence of the correct deletion alleles by colony PCR [20] and primers 597 and 1546 for Δ(*amrR-amrAB-oprA*)::*FRT*-*nptII*-*FRT*. An unmarked Δ(*amrR-amrAB-oprA*) mutation was obtained after Flp recombinase- mediated excision of the *nptII* marker using pFlpe2 [20]. The presence of the deletion allele was verified by phenotypic (Gm susceptibility) and genotypic (PCR with primers 1581 and 1584) analyses.

### Fosmid library construction and screening

Genomic DNA was extracted from strain 708a using the QiAmpDNA Mini Kit (Qiagen, Valencia, CA). Fosmids containing ∼40 kb inserts were isolated using the CopyControl Fosmid Library Production Kit following manufacturer's instructions (Epicentre, Madison, WI). Approximately 1,200 Cm^r^ resistant colonies were pooled in groups of 30 (designated pools A–Z and 1–11), grown overnight in Cm containing medium, induced to high copy number and fosmid DNA was extracted using the QIAprep Mini-spin kit (Qiagen). Fosmid DNA from the 30 pools were screened by PCR using primers 1742 and 1743, and PCR products were obtained from 5 pools. DNA from these pools was transformed into *E. coli* DH5α and single colonies were screened for the presence of the correct clones by PCR using primers 1742 and 1743. DNA was extracted from these clones and sequenced with primers 1774 and 1779 which anneal in the fosmid backbone, as well as 1742 which anneals in the insert. Sequences obtained with primers 1774 and 1779 were BLAST searched against genome sequences of *B. pseudomallei* strains K96243, 1710b, 1106a and 668.

### Next Gen Sequencing and data analysis

The genome of strain 708a was sequenced using a short “read” technology to detect missing genes relative to reference genomes. Five µg of DNA from *B. pseudomallei* strain 708a was sheared into approximately 175 bp fragments using air nebulization. A genomic library was then constructed following standard protocols from Illumina, Inc. (San Diego, CA). The library was sequenced on an Illumina Genome Analyzer (GA) using a single read sequencing method. Image analysis for base calling and alignments followed protocols of Craig *et al.* [25]. Genomic sequencing data (42 bp reads) for strain 708a were aligned against the K96243 and MSHR668 (data not presented) reference genomes using the Illumina GA software. The aligned reads were then visualized using the software program SolScape (Beckstrom-Sternberg et al., manuscript in preparation). Genomic regions with no reads were interpreted as missing from the sequenced genome.

### Isolation of gentamicin resistant mutants

Gentamicin resistant derivatives of strains 2188a and 3799a were isolated in several steps. First, the strains were grown overnight at 37°C in LSLB medium containing 8 µg/ml Gm. The bacteria were then diluted into fresh LSLB medium containing 16 µg/ml Gm, followed by outgrowth at 37°C. The selection steps were repeated using LSLB medium containing 32, 64 and 128 µg/ml Gm. Similar selection steps were performed with 708a except that lower Gm concentrations of 2, 4, 8 and 16 µg/ml were employed.

### Antimicrobial susceptibility testing

Minimal inhibitory concentrations (MICs) were determined in Mueller-Hinton broth from Becton Dickinson (Franklin Lakes, NJ) by the two-fold broth microdilution technique following Clinical and Laboratory Standards Institute guidelines [26]. The MICs were recorded after incubation at 37°C for 15 to 16 h.

### Animal infection experiments

Ethics Statement: All animal procedures were performed using standard protocols and according to guidelines approved by the Colorado State University BioSafety Committee and the Colorado State University Animal Care and Use Committee. For animal infection experiments, *B. pseudomallei* strains were grown in LB medium to saturation by overnight incubation at 37°C with aeration. Glycerol was added to a final concentration of 15% and cell suspensions were stored at −80°C until ready for use. Inocula for *in vivo* infections were prepared by thawing and diluting the frozen bacterial stocks in sterile phosphate buffered saline (Sigma-Aldrich). Female BALB/c mice between 6–8 weeks of age were used for infection studies (Jackson Laboratories, Bar Harbor, ME). Mice were housed under pathogen-free conditions, and provided sterile water and food *ad libitum*. All animal infections were done using intranasal (i.n.) inoculation. Mice were anesthetized by intraperitoneal injection of 100 µg/g body weight of ketamine (Fort Dodge Animal Health, Overland Park, KS) and 10 µg/g body weight of xylazine (Ben Venue Laboratories, Bedord, OH). For all infections, the desired inoculum of *B. pseudomallei* was suspended in phosphate buffered saline. The 20 µl inoculum volume was delivered i.n, with the dose split evenly between both nostrils. At the completion of challenge studies, animals were humanely euthanized, according to study endpoints approved by the Animal Care and Use Committee at Colorado State University.

## Results and Discussion

### Aminoglycoside and macrolide susceptible isolates show reduced or absent AmrAB-OprA expression

**Figure 1:**
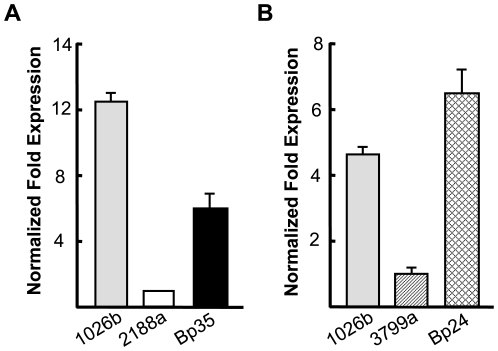
*amrB* transcript levels in gentamicin susceptible and resistant strains. mRNA levels in LSLB without antibiotics-grown late-log cultures of the indicated strains were determined with an *amrB*-specific primer set. Data were normalized using the 23S rRNA gene as the housekeeping control. *amrB* transcript levels were determined A in strain 2188a and its gentamicin resistant derivative Bp35 and B in strain 3799a and its gentamicin resistant derivative Bp24. Relative quantifications were performed using 2188a and 3799a, respectively.

In agreement with previously published results, the aminoglycoside and macrolide susceptibility patterns of strains 708a, 2188a and 3799a isolated from human patients with various disease manifestations and clinical outcome (**Table 2**) were similar to those observed with the AmrAB-OprA deficient strain DD503 (**Table 3**). Quantitative real-time PCR was therefore used to assess *amrAB-oprA* expression relative to strain 1026b, which is known to constitutively express this efflux pump. No *amrB* transcripts were detected in strains 708a and Δ(*amrAB-oprA*) strain DD503, and *amrB* transcript levels were significantly lower in 2188a and 3799a than those measured in 1026b (**Fig. 1**). As in our hands 2 to 3 fold differences in mRNA levels determined by qRT-PCR make the difference between low- and high-level RND pump-mediated resistance, these data support the notion that the aminoglycoside and macrolide susceptibilities of strains 708a, 2188a and 3799a are due to reduced or lack of AmrAB-OprA efflux pump expression.

**Table 2:** *B. pseudomallei* strains: origins, properties and clinical details.

Strain	Isolation Date	Clinical Details	Gentamicin MIC^a^
708a	30.8.90	32 year old male; 21 days fever and 14 days abdominal pain. No risk factors for melioidosis. Splenic abscess as single infectious site. Splenectomy required to control infection. Treated with intravenous ceftazidime followed by oral doxycycline. Survived.	0.5 µg/ml
2188a	18.12.98	22 year old male rice farmer; 14 days fever, cough, sputum, swollen left knee. Known diabetic. Bacteremic with lung and joint involvement. Treated with joint washout and intravenous amoxicillin/clavulanic acid. Developed respiratory failure and died the day after admission.	0.5 µg/ml
3799a	12.12.05	66 year old female rice farmer; 15 days cough, breathlessness, sputum. History of chronic renal failure. Bacteremic with lung and renal involvement. Treated with ceftazidime. Died from septic shock 4 days after admission.	1 µg/ml

a MIC determinations were performed in Thailand using the E-test.

**Table 3:** Antibiotic susceptibilities of *B. pseudomallei* strains.

Strain	Known Genotype	MIC (µg/ml) for:					
		Gm^a^	Str	Spc	Ery	Cla	Cli
1026b	Wild-type	256	1024	512	128	64	>1024
DD503	1026b with Δ(*amrR-amrAB-oprA*)	2	ND^b^	64	8	4	>1024
708a		1	8	32	16	16	>1024
2188a		1	8	32	16	32	>1024
3799a		2	8	64	16	16	>1024
Bp24	Gm^r^ derivative of 3799a	>1024	1024	256	64	16	>1024
Bp35	Gm^r^ derivative of 2188a	>1024	>1024	>1024	256	512	>1024
Bp66	Low level Gm^r^ derivative of 708a	32	8	16	4	16	>1024
Bp187	Bp24 with Δ(*amrR-amrAB-oprA*)	2	16	128	16	16	>1024
Bp202	Bp187::mini-Tn*7*T-LAC^c^	4	32	128	8	16	>1024
Bp194	Bp187::mini-Tn*7*T-LAC-*amrA* ^+^ *B* ^+^-*oprA* ^+c^	>1024	>1024	>1024	256	512	>1024
Bp192	Bp35 with Δ(*amrR-amrAB-oprA*)	2	16	128	16	16	>1024
Bp201	Bp192::mini-Tn*7*T-LAC^c^	4	32	128	8	16	>1024
Bp200	Bp192::mini-Tn*7*T-LAC-*amrA* ^+^ *B* ^+^-*oprA* ^+c^	>1024	>1024	>1024	256	256	>1024

a Cla, clarithromycin; Cli, clindamycin; Ery, erythromycin; Gm, gentamicin; Spc, spectinomycin; Str, streptomycin.

b ND, not done; DD503 is streptomycin resistant because of a chromosomal *rpsL* mutation.

c The mini-Tn*7* elements are integrated at the *glmS2*-associated Tn*7* attachment site [20]. MIC values were determined in cells grown in the presence of 1 mM isopropyl-β-D-thiogalactopyranoside.

### Gentamicin resistant derivatives of 2188a and 3799a, but not 708a, express AmrAB-OprA

As we were able to PCR amplify the 5′ and 3′ regions of the *amrAB-oprA* operon from strains 2188a and 3799a, but not 708a (data not shown), we suspected that this operon was absent from 708a and present but expressed at low levels 2188a and 3799a. To test this notion, we attempted to isolate Gm resistant derivatives of these strains. Highly (MIC>1024 µg/ml) Gm^r^ derivatives, e.g. Bp35 and Bp24, were readily obtained with strains 2188a and 3799a, but not with 708a (e.g. Bp66) (**Table 3**). Moreover, the Gm^r^ 2188a and 3799a derivatives Bp35 and Bp24 became simultaneously resistant to other aminoglycosides and macrolides and their antibiotic susceptibility profiles resembled that of AmrAB-OprA expressing strain 1026b (**Table 3**). In contrast, the moderately (MIC 32 µg/ml) Gm^r^ derivative of 708a (Bp66) did not simultaneously become resistant to other aminoglycosides and erythromycin. None of the strains tested exhibited altered clindamycin resistance. Clindamycin is a good substrate of BpeAB-OprB but not AmrAB-OprA (T. Mima and H. Schweizer, unpublished data). Consistent with these observations, significantly increased *amrB* transcript levels were detected in Bp24 and Bp35 (**Fig. 1**, panels **A** and **B**), but not Bp66 (not shown). Deletion of *amrAB-oprA* from Bp24 and Bp35 resulted in loss of aminoglycoside and macrolide resistance which could be complemented back to wild-type levels by a chromosomally integrated mini-Tn*7* expressing *amrA^+^B^+^-oprA^+^* (**Table 3**). Together, these results indicate that the *amrAB-oprA* operon is absent from 708a and present, but not expressed in sufficient levels in strains 2188a and 3799a to confer aminoglycoside and macrolide resistance.

### Lack of AmrAB-OprA expression in 2188a and 3799a is not due to mutations in the *amrAB-oprA* regulatory region

To assess whether lack of *amrAB-oprA* expression in strains 2188a and 3799a is due to mutations in the operon's regulatory region, the *amrR-amrA* intergenic region was amplified with primers 1601 and 1602 and sequenced. These analyses revealed that the sequence of the *amrR-amrA* intergenic regions of strains 2188a and 3799a and their Gm^r^ derivatives Bp35 and Bp24 were identical (data not shown). Furthermore, amplification of the *amrR* coding sequences from 2188a and 3799a and their Gm^r^ derivatives Bp35 and Bp24 with primers 1599 and 1600 did not reveal any mutations in *amrR*. In summary, these data revealed that i) lack of AmrAB-OprA expression in 2188a and 3799a was not caused by mutations in the *amrAB-oprA* regulatory region and ii) increased *amrAB-oprA* expression in Gm^r^ derivatives Bp24 and Bp35 was not due to promoter-up mutations or other *amrR* mutations. Rather, the data suggest that AmrAB-OprA expression is governed by a yet unidentified transcription factor or other positive regulatory mechanism(s). It is well known that efflux pump operon expression in other bacteria is governed by local as well as global mechanisms (reviewed in [27]). For instance, *mexAB-oprM* operon expression in *P. aeruginosa* is under control of the local MexR repressor [28], as well as other mechanisms including the ArmR anti-repressor encoded by a gene elsewhere on the chromosome [29].

### Strain 708a contains a large deletion on chromosome 1

Results of PCR and qRT-PCR analysis were consistent with the notion that the *amrAB-oprA* operon was missing from strain 708a. Using the 1710b chromosome 1 sequence as a guide, primer sets were designed to amplify ∼500 bp fragments in the *amrAB-oprA* containing region of chromosome 1. Results of this primer walking strategy identified a correct PCR product obtained with primer set 1742 and 1743 designed to amplify sequences located ∼5 kb upstream of *amrR*. However, no PCR products were obtained with primers designed to sequences located up to 65 kb downstream of *oprA*. These data were consistent with the presence of a large (>70 kb) deletion on chromosome 1 encompassing *amrAB-oprA*. To determine the deletion boundaries, a fosmid library was constructed using 708a chromosomal DNA. By PCR amplification, several fosmids containing DNA previously located ∼5 kb upstream of *amrR* were identified. Sequence analyses of both fosmid-chromosomal DNA boundaries and BLAST analyses using four *B. pseudomallei* genomes revealed the same open reading frames (ORFs) at the respective junctions, BURPPS1710b_2037 (or its respective homolog in other genomes) and BURPPS1710b_2160 (or its respective homolog in other genomes). A series of primers was designed to determine the sequence adjacent to the primer 1742 binding site. The sequence was aligned to that of 1710b and revealed a fusion of ORFs *BURPPS1710b_2155* and *BURPPS1710b_2054*. We interpreted this to mean that compared to 1710b, the 708a sequence was missing nucleotides 2,219,259–2,359,936 (or ∼141 kb) from chromosome 1, including *amrAB-oprA*.

**Figure 2:**
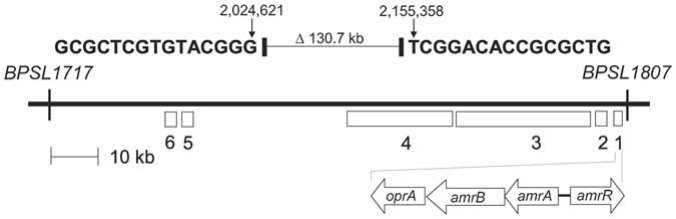
Extent of chromosome 1 deletion in strain 708a compared to K96243. 708a contains a deletion fusing the bold sequences of *BPSL1717* and *BPSL1807*, respectively. Some notable genes and gene clusters present in K96243 but missing from 708a are: 1 *amrR-amrAB-oprA*; 2 a three gene operon (*BPSL1801-BPSL1800-BPSL1799*) encoding a putative type-1 fimbrial protein along with its outer membrane usher protein and chaperone; 3 the 13 gene malleobactin biosynthetic gene cluster and its extracytoplasmic sigma factor MbaS defined by *mbaF-fmtA-mbaA-mbaI-mbaJ-mbaE*-*BPSL1781-BPSL1782-BPSL1783-BPSL1784-BPSL1785-BPSL1786-mbaS*; 4 a cluster of 18 genes (*BPSL1755*-*BPSL1773*) encoding a putative aerobic (or late cobalt insertion) vitamin B_12_ biosynthetic pathway with an embedded gene (*BPSL1763*) encoding a putative exported chitinase; 5 *arcD (BPSL1742)* and *arcABC* (*BPSL1743*-*BPSL1744*-*BPSL1745*) coding for the arginine deiminase pathway; and 6 a two gene cluster (*BPSL1732*-*BPSL1731*) coding for a putative methyl-accepting chemotaxis citrate transducer and chemotaxis protein CheW2, respectively. Strain 1710b contains an additional 10 kb of DNA in this region.

**Figure 3:**
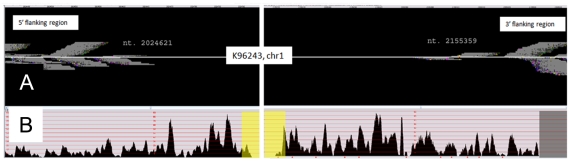
Large deletion verification in chromosome 1 of strain 708a by whole genome sequencing. Genomic sequencing data from strain 708a were aligned against the K96243 reference genome. Panel A shows the read density near positions 2,024,621 and 2,155,359 on chromosome 1. Panel B shows the 708a read density across the ∼4.5 Kb flanking the deletion in chromosome 1 of strain K96243. The yellow highlighted region in panel B marks a ∼130.7 Kb region with a near-zero read coverage, which correspond to the panel A coordinates. This lack of reads is strong evidence for deletion of the entire region in strain 708a.

When compared to other strains, the extent of the deletion varied by approximately ±10 kb based on sequence from strains used as comparators. For example, when compared to K96243 the deletion is ∼131 kb (**Fig. 2**). The deletion was further confirmed by: i) PCR amplification using primers 1797 and 1801 and DNA sequence analysis of a 1.1 kb chromosomal DNA fragment from 708a genomic DNA containing the predicted deletion junction; and ii) short read whole genome sequencing of the 708a genome (**Fig. 3**).

### Genes contained within the large deletion present in 708a chromosome 1

**Figure 4:**
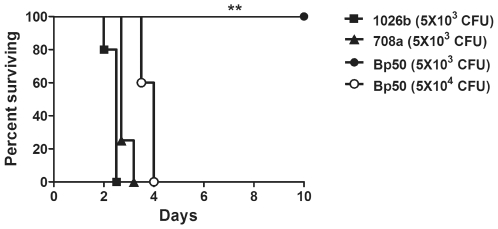
Strain 708a is fully virulent in an acute murine melioidosis infection model. BALB/c mice (*n* = 4–5 mice) were infected intranasally with 5×10^3^ CFUs of 1026b ▪, 5×10^3^ CFUs of strain 708a ▴, and 5×10^3^ • or 5×10^4^ ○ colony forming units of the isogenetic Δ(*amrRAB-oprA*) 1026b derivative Bp50. Statistical differences in survival times were determined by Kaplan-Meier curves followed by log-rank test. The Bonferroni corrected threshold was applied and comparisons with *p *<0.017 were considered significant. (**, *p *<0.01 for strain 1026b vs. Bp50 (5,000 CFU) and 708a vs. Bp50 (5,000 CFU). Data are representative of 2 independent experiments.

Because of the more thorough and detailed annotation of the published K96243 genome we decided to use it to assess key genes missing from *B. pseudomallei* strain 708a. According to K96243 coordinates, 708a is missing nucleotides 2,024,622 to 2,155,357 fusing the *BURPPS1710b_2155* and *BURPPS1710b_2054* equivalents *BPSL1717* and *BPSL1807* (**Fig. 2**). In K96243, as well as 1710b and other *B. pseudomallei* strains, this >90 gene region not only contains *amrAB-oprA* but several other genes that may be pertinent to this bacterium's physiology and pathogenesis (**Table 4**). First, this deleted region contains the 13 gene malleobactin biosynthetic gene cluster and its extracytoplasmic sigma factor MbaS defined by *mbaF-fmtA-mbaA-mbaI-mbaJ-mbaE*-*BPSL1781-BPSL1782-BPSL1783-BPSL1784-BPSL1785-BPSL1786-mbaS* [30]. Malleobactin is a hydroxamate siderophore that is analogous to the same genes in *Pseudomonas aeruginosa* pyoverdine [31] and *B. cepacia* ornibactin [32]. Pyoverdine is essential for infection and full virulence of *P. aeruginosa*, as assessed in several different experimental models [33], along with biofilm formation [34]. Similarly, *B. cepacia* mutants defective in ornibactin synthesis showed significantly reduced virulence [32]. However, in the case of 708a, despite missing the entire malleobactin biosynthetic gene cluster and exhibiting overall greatly reduced siderophore synthesis (as assessed by growth on Chrome azurol S plates) [30],[35] (data not shown), the 708a stain was still able to cause severe illness in the infected human from which it was isolated (**Table 2**). Moreover, strain 708a was also fully virulent in our acute inhalational challenge model in mice (**Fig. 4**). Thus, it is possible that malleobactin may not play the same crucial role in infection and virulence that the *P. aeruginosa* pyoverdine siderophore does. Alternatively, *B. pseudomallei* is known to synthesize other iron transport systems, including a pyochelin siderophore and heme-hemin receptor and transporter [30],[36], and thus 708a may utilize these alternative pathways for iron transport. Second, immediately adjacent to the malleobactin biosynthetic genes is a cluster of 18 genes (*BPSL1755*-*BPSL1773*) encoding a putative aerobic (or late cobalt insertion) vitamin B_12_ biosynthetic pathway [37]. Vitamin B_12_ is a known cofactor for numerous enzymes mediating methylation, reduction and intramolecular rearrangements. Why this pathway is dispensable for growth in 708a is not known. However, some bacteria are known to possess an alternative anaerobic (or early cobalt insertion) pathway [37]. Third, the deletion in 708a encompasses the genes *arcD (BPSL1742)* and *arcABC* (*BPSL1743*-*BPSL1745*) coding for the arginine deiminase pathway. In *P. aeruginosa*, this pathway provides for ATP synthesis under anaerobic conditions in the absence of exogenous electron acceptors provided that arginine is present in the growth medium [38]. In this context it is worthy of note that 708a was isolated from a splenic abscess and abscesses are generally considered to provide a mixed aerobic-anaerobic environment [39],[40]. If 708a was truly able to grow under anaerobic conditions, then 708a must be capable of utilizing alternative pathways for energy generation under anaerobic conditions. This alternate pathway presumably would require nitrate as *B. pseudomallei* was shown to be capable of growing anaerobically only in the presence of arginine and nitrate [41].

**Table 4:** K96243 gene equivalents contained within the 708a chromosome 1 deletion.

Locus Tag or Gene	Putative or Known Function
*BPSL1717*	Hypothetical protein
*BPSL1718*	Hypothetical protein
*BPSL1719*	Putative kinase
*BPSL1720*	Putative argininosuccinate lyase
*BPSL1721*	Putative argininosuccinate synthase
*BPSL1722*	Putative formyl transferase
*BPSL1723*	Hypothetical protein
*BPSL1724*	Putative histidinol-phosphate aminotransferase
*BPSL1725*	Hypothetical protein
*BPSL1726*	Hypothetical protein
*BPSL1727*	Putative non-ribosomal peptide synthase (thioesterase domain)
*BPSL1727*	Putative non-ribosomal peptide synthase (thioesterase domain)
*BPSL1728*	Putative exported porin
*BPSL1729*	Putative AraC-family transcriptional regulator
*BPSL1730*	Putative transmembrane protein
*BPSL1731*	Chemotaxis protein CheW2
*BPSL1732*	Putative methyl-accepting chemotaxis citrate transducer
*BPSL1733*	Hypothetical protein
*BPSL1734*	Acyl-CoA synthase
*BPSL1735*	Putative transport system membrane protein
*BPSL1736*	Putative methyltransferase
*BPSL1737*	Putative ABC transport system, exported protein
*BPSL1738*	Putative ABC transport system, membrane protein
*BPSL1739*	Putative ABC transport system, ATP-binding protein
*BPSL1740*	Putative ABC transport system, membrane protein
*BPSL1741*	Hypothetical protein
*arcD*	Arginine/ornithine antiporter
*arcA*	Arginine deiminase
*arcB*	Ornithine carbamoyltransferase
*arcC*	Carbamate kinase
*BPSL1746*	Short chain dehydrogenase
*BPSL1747*	Hypothetical protein
*BPSL1748*	Putative LysR-family transcriptional regulator
*BPSL1749*	Putative glutathione *S*-transferase
*BPSL1750*	Putative MarR-family transcriptional regulator
*BPSL1751*	Putative amino-acid transport-related exported protein
*BPSL1752*	Putative MarR-family regulatory protein
*BPSL1753*	Putative transport-related membrane protein
*BPSL1754*	Putative lipoprotein
*BPSL1755*	Precorrin-4 C11-methyltransferase
*BPSL1756*	Precorrin-6× reductase
*BPSL1757*	Cobalt-precorrin-6A synthase
*BPSL1758*	Precorrin-6Y C5,15-methyltransferase
*BPSL1759*	Putative oxidoreductase
*BPSL1760*	Precorrin-8× methylmutase
*BPSL1761*	Precorrin-2 methyltransferase
*BPSL1762*	Precorrin-3b C17-methyltransferase
*BPSL1763*	Putative exported chitinase
*BPSL1764*	Hypothetical protein
*BPSL1765*	Putative carboxylesterase
*BPSL1766*	Hypothetical protein
*BPSL1767*	Putative magnesium chelatase protein
*BPSL1768*	Cobaltochelatase
*BPSL1769*	Putative cobalamin biosynthesis-related protein
*BPSL1770*	High-affinity nickel transport protein
*BPSL1771*	Cobalamin biosynthesis protein CbiG
*BPSL1772*	Cob(I)yrinic acid a,c-diamide adenosyltransferase
*BPSL1773*	Cobyrinic acid A,C-diamide synthase
*mbaF*	Putative N^5^-hydroxyornithine transformylase^1^
*fmtA*	Malleobactin receptor
*mbaA*	Putative L-ornithine-N^5^-oxygenase
*mbaI*	Putative non-ribosomal peptide synthase
*mbaJ*	Putative non-ribosomal peptide synthase
*mbaE*	Similar to *P. aeruginosa pvdE* (ABC transporter)
*BPSL1780*	Hypothetical protein
*BPSL1781*	Putative periplasmic iron-binding protein
*BPSL1782*	Putative ferric iron reductase
*BPSL1783*	Putative iron transport-related membrane protein
*BPSL1784*	Putative iron transport-related ATP-binding protein
*BPSL1785*	Hypothetical protein (similar to *syrP* from *Streptomyces verticillus*)
*BPSL1786*	Hypothetical protein (similar to *mbtH* from *Mycobacterium tuberculosis*)
*mbaS*	MbaS, extracytoplasmic sigma factor
*BPSL1788*	Pseudogene
*BPSL1789*	Short chain dehydrogenase
*BPSL1790*	Putative zinc-binding dehydrogenase
*BPSL1791*	Hypothetical protein
*BPSL1792*	Hypothetical protein
*BPSL1793*	Putative sugar-binding exported protein
*BPSL1794*	Putative AraC-family transcriptional regulator
*BPSL1795*	Hypothetical protein
*BPSL1796*	Hypothetical protein
*BPSL1797*	Putative ABC transport system, membrane protein
*BPSL1798*	Hypothetical protein
*BPSL1799*	Putative fimbrial chaperone
*BPSL1800*	Putative outer membrane usher protein precursor
*BPSL1801*	Putative type-1 fimbrial protein
*BPSL1802*	OprA multidrug efflux outer membrane channel protein
*BPSL1803*	AmrB multidrug efflux system transporter protein
*BPSL1804*	AmrA multidrug efflux system membrane fusion protein
*BPSL1805*	AmrR TetR family regulatory protein
*BPSL1806*	Subfamily M23B unassigned peptidase
*BPSL1807*	Putative amino acid transport system, membrane protein

1 Annotation of *BPSL1774* (*mbaF*) through *BPSL1787* (*mbaS*) according to Alice et al. [30].

Fourth, other noteworthy genes covered by the deletion include i) a three gene operon (*BPSL1801-BPSL1799*) encoding a putative type-1 fimbrial protein along with its outer membrane usher protein and chaperone; ii) a two gene cluster (*BPSL1732*-*BPSL1731*) coding for a putative methyl-accepting chemotaxis citrate transducer and chemotaxis protein CheW2, respectively; and iii) a putative exported chitinase (*BPSL1763*).

### Genes missing from the 131 kb deletion are not present elsewhere on the chromosome

To assess whether the aforementioned genes were indeed absent from the chromosome we performed i) whole genome sequencing and ii) PCR analysis of selected genes.

Genomic alignments were performed to compare 708a data with two *B. pseudomallei* reference genomes: strains K96243 and MSHR668. The 42 bp reads had an average density of 24× and covered 93.3% (chromosome 1) and 96.9% (chromosome 2) of the reference genomes. The notable exception to this coverage was a ∼130.7 Kb region corresponding to positions 2,024,621 and 2,155,359 in chromosome 1 of the K96243 genome (**Fig. 3**). Nearly zero reads aligned to this region indicating that the 708a strain does not contain any of these genes. While these data do not discern gene order or chromosomal linearity between 708a and the reference genomes, this does represent a comprehensive query and argues that the genes in this region are not present anywhere in the 708a genome. If homologous genes existed elsewhere in the 708a genome, they would have generated short reads that would have aligned with this region. The short read data are available online at http://www.mggen.nau.edu/MGGen_research.html.

**Figure 5:**
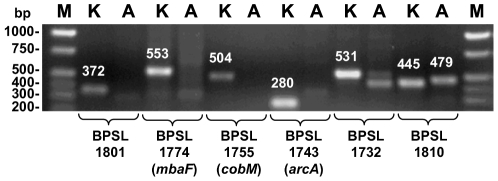
Deleted genes are absent from the 708a genome. PCR was performed with genomic DNA isolated from K strain K96243 or A strain 708a with gene-specific primers. These included 2037 & 2038 for *BPSL1801*, 2035 & 2036 for *BPSL1774*, 2033 & 2034 for *BPSL1755*, 1954 & 1955 for *BPSL1743*, 2031 & 2032 for *BPSL1732*, and 1742 & 1743 for *BPSL1810*. PCR products were separated on a 1% agarose gel and stained with ethidium bromide. Sizes of the expected PCR fragments (in bp and based on K96243 genomic sequence) are indicated above the respective bands. Gene annotations are according to K96243 and gene names, where known, are in parentheses. Lanes M contained the Hi-Lo DNA ladder (Minnesota Molecular, Minneapolis, MN) and the sizes of pertinent fragments are indicated on the left.

Because whole genome sequence coverage was not 100% for both chromosomes, we performed PCR analysis for selected genes using gene-specific primers designed for amplification of the corresponding K96243 sequences (**Fig. 5**). PCR analysis showed the expected DNA fragments with genomic DNA templates from K96243 but not with 708a templates. The identities of the amplified DNA fragments were verified by DNA sequence analyses which also confirmed minor bands visible in some PCR reactions from 708a templates as non-specific amplification products. As a positive control, we amplified a fragment from the *BPSL1809-BPSL1810* region using primers 1742 and 1743. This region is present in both 708a and K96243. The 445 bp amplicon derived from K96243 DNA consists of 243 bp from BPSL1810 and 202 bp from the *BPSL1809-BPSL1810* intergenic region. The corresponding fragment obtained with 708a DNA is slightly larger (479 bp) because of several insertions in the *BPSL1809-BPSL1810* intergenic region.

In summary, these findings provide some insight into the physiology and pathogenesis of *B. pseudomallei*. However, because 708a grows normally in rich and minimal laboratory media under aerobic conditions, is fully virulent in an acute murine melioidosis model and caused human melioidosis, the genes affected by the deletion must be dispensable at least under the *in vitro* and *in vivo* conditions encountered during laboratory studies and splenic abscess disease during human infection caused by lone presence of 708a. This scenario is likely as simultaneous infection with more than one strain is uncommon in human melioidosis [42].

### Concluding remarks

The clinical diagnosis of *Burkholderia pseudomallei* still relies on culture which is most commonly performed using selective Ashdown's agar whose main selective ingredient is gentamicin. The majority of *B. pseudomallei* strains grow on this medium because of their intrinsic resistance to aminoglycosides mediated by the AmrAB-OprA efflux pump. At least 1 in 1,000 clinical isolates in NE Thailand are susceptible to aminoglycosides and such isolates are obviously missed by using Ashdown's diagnostic agar. The actual number of aminoglycoside susceptible strains may thus be higher. Our results confirm that the aminoglycoside and macrolide susceptibility of rare clinical isolates is indeed due to reduced or lack of expression of the *amrAB-oprA* efflux pump operon, as previously suggested but not proven [16]. Even though BpeAB-OprB was previously implicated to contribute to aminoglycoside and macrolide resistance in strain KHW [14], we now know that this pump does not confer aminoglycoside resistance in 1026b (T. Mima and H. Schweizer, unpublished observations), a strain isolated in the same hospital as 708a. BpeAB-OprB is only expressed at very low levels in wild-type strains which may explain the low levels of erythromycin resistance observed in 708a, 2188a and 3799a in the absence of AmrAB-OprB. This notion is supported by the observation that all strains analyzed in this study exhibit clindamycin resistance. Clindamycin is a good substrate of BpeAB-OprB but not AmrAB-OprA (T. Mima and H. Schweizer, unpublished data). As expected, qRT-PCR analyses showed only low-level BpeAB-OprB expression in these strains (data not shown). Though strain 708a contains a large deletion encompassing several gene clusters encoding potential virulence factors and genes required for growth under anaerobic conditions, these genes may either be dispensable for *in vitro* and *in vivo* growth or this strain compensates for them by expressing similar functions from another set of genes. The latter notion may be supported by the observation that the genetically engineered 1026b AmrAB-OprA mutant derivative Bp50 shows reduced virulence in the murine melioidosis model whereas 708a missing these genes is as virulent as 1026b (**Fig. 4**). We do not know the factors, if any, that led to selection of strains missing or lacking expression of AmrAB-OprA. Further experiments aimed at addressing some of these issues at the molecular level are facilitated by availability of the nearly complete 708a sequence and tools that allow genetic manipulation of this strain. Lastly, because 708a is fully virulent in the murine melioidosis model, yet very susceptible to aminoglycosides, this strain may be a natural candidate for genetic manipulation experiments that use Select Agent compliant antibiotics for selection, such as gentamicin [20], kanamycin [20], spectinomycin/streptomycin [43] and nourseothricin [44] selection markers, and validates the use of laboratory-constructed Δ(*amrAB-oprA*) mutants in such experiments [13],[20].
